# DotAligner: identification and clustering of RNA structure motifs

**DOI:** 10.1186/s13059-017-1371-3

**Published:** 2017-12-28

**Authors:** Martin A. Smith, Stefan E. Seemann, Xiu Cheng Quek, John S. Mattick

**Affiliations:** 1 RNA Biology and Plasticity Group, Garvan Institute of Medical Research, 384 Victoria Street, Sydney, NSW 2010 Australia; 2 St Vincent’s Clinical School, Faculty of Medicine, UNSW Australia, Sydney, NSW 2010 Australia; 3 Center for non-coding RNA in Technology and Health (RTH), University of Copenhagen, Groennegaardsvej 3, Frederiksberg, 1870 Denmark; 4 Department of Veterinary and Animal Sciences, Faculty of Health and Medical Sciences, University of Copenhagen, DK-1870, Frederiksberg, Denmark

**Keywords:** Functions of RNA structures, RNA structure clustering, Machine learning, RNA–protein interactions, Functional genome annotation, Regulation by non-coding RNAs

## Abstract

The diversity of processed transcripts in eukaryotic genomes poses a challenge for the classification of their biological functions. Sparse sequence conservation in non-coding sequences and the unreliable nature of RNA structure predictions further exacerbate this conundrum. Here, we describe a computational method, DotAligner, for the unsupervised discovery and classification of homologous RNA structure motifs from a set of sequences of interest. Our approach outperforms comparable algorithms at clustering known RNA structure families, both in speed and accuracy. It identifies clusters of known and novel structure motifs from ENCODE immunoprecipitation data for 44 RNA-binding proteins.

**Electronic supplementary material:**

The online version of this article (doi:10.1186/s13059-017-1371-3) contains supplementary material, which is available to authorized users.

## Background

As genomic technologies progress, an ever-increasing number of non-protein-coding RNAs (ncRNAs) are being discovered. Long non-coding RNAs (lncRNAs) are of particular interest for functional genome annotation given their abundance throughout the genome. So far, few lncRNAs have been functionally characterised, and those that have seem to be involved in regulation of gene expression and epigenetic states [1, 2]. Understanding the molecular mechanisms underlying the biological functions of lncRNAs – and how they are disrupted in disease – is required to improve the functional annotation of the human genome.

Many ncRNAs lack sequence conservation, in contrast to protein-coding genes. Most small ncRNAs have well characterised secondary and tertiary structures, as evidenced in Rfam, the largest collection of curated RNA families (2588 families as of version 12.2 [3]). In contrast, determining the structural features of lncRNAs is a complex problem given their size and, in general, faster evolutionary turnover. These challenges have raised doubts concerning the prevalence of functional structural motifs in lncRNAs [4, 5], despite evolutionary and biochemical support for conserved base pairing interactions [6–8]. Nonetheless, the higher-order structure of RNA molecules is an essential feature of ncRNAs, which can be used for their classification and the inference of their biological function.

We, and others, hypothesise that lncRNAs act as scaffolds for the recruitment of proteins and assembly of ribonucleoproteins (RNPs), mediated by the presence of modular RNA structures, akin to the domain organisation of proteins [6, 9–14]. Protein-interacting regions of lncRNAs are likely to contain a combination of sequence and structure motifs that confer binding specificity, which may be present in multiple target transcripts. For example, there is evidence that sequence and structure components of transposable elements, which are frequent in lncRNAs [15, 16], have been co-opted into mammalian gene regulatory networks [17, 18]. Identifying and annotating the genomic occurrence of homologous RNA structure motifs from sets of biologically related sequences will improve our understanding of the structure–function relationship of lncRNAs and the molecular mechanisms underlying their regulatory features. Resolving this challenge can be beneficial for the analysis of high-throughput RNA sequencing experiments that measure how RNAs interact with other molecules, such as cross-linked RNA immunoprecipitation and RNAse footprinting methodologies.

The identification of RNAs with similar functions involves comparing both their primary sequence and higher-order structures simultaneously. However, sequence-based methods to identify common structural features perform poorly when sequence identity falls below 60 % [19]. Hence, methods are needed that find structural similarity independent from sequence conservation and freed from single RNA secondary structure predictions. The Sankoff algorithm resolves the optimal sequence-structure alignment of two RNAs [20], but its computational complexity limits its practicality. Its most comparable implementation, FoldAlign, employs a minimum free energy-based strategy with pruning of the associated dynamical programming matrix [21, 22]. Alternative strategies often employ pre-calculated secondary structure probability distributions (thermodynamically equilibrated canonical ensembles) for each sequence [23]. These can substantially speed up the calculation of structure-based alignments [24], of which there are many variants. The programs PMcomp [24], LocaRNA [25] and ProbAlign [26] use the pre-computed base pair probability matrices of two sequences and score the alignment based on the notion of a common secondary structure. The sequence-structure alignment problem is reduced to a two-dimensional problem in RNApaln [27] and StrAL [28], which derive probabilities for individual bases (such as the probability of being unpaired) from all base pairing probabilities. These methods all fail to consider explicitly suboptimal structures in the alignment. The pairwise alignment of entire base pairing probability matrices (RNA *dot plots*) was first introduced by CARNA [29, 30], which iteratively improves alignments using a constraint programming technique implementing a branch and bound scheme.

These pairwise RNA structure alignment algorithms can be used to identify clusters of homologous RNA structure motifs from a set of sequences of interest. Will et al. first showed that a (dis)similarity matrix can be constructed from all-vs-all pairwise RNA structure alignments with the pairwise alignment tool LocaRNA, identifying known and novel groups of homologous RNAs using hierarchical clustering [25]. However, this strategy involves applying a subjective threshold to the resulting dendrogram to extract structurally related sequences. Alternative approaches to all-vs-all pairwise comparisons for RNA structure clustering include NoFold, which clusters query sequences based on their relative similarity to a panel of reference structure motif profiles [31], and GraphClust, an alignment-free approach that decomposes RNA structures into graph-encoded features [32]. RNAscClust, an extension of GraphClust, utilises the evolutionary signatures of RNA structures (when available) as an additional classification feature [33].

Here, we describe a computational pipeline for the identification and clustering of homologous RNA structures from a large set of query sequences. At its core lies DotAligner, a heuristic pairwise sequence alignment algorithm that considers the ensemble of base pair probabilities for each queried sequence. We benchmark the performance of DotAligner with other pairwise RNA structure alignment algorithms through several iterations of a stochastic sampling strategy across all Rfam seed alignments, highlighting the speed and accuracy of our method. We combine DotAligner with density-based clustering for the unsupervised identification of RNA structure motifs, which can identify both known Rfam families and novel RNA structure motifs from ENCODE enhanced cross-linked immunoprecipitation (eCLIP) data. Finally, we exemplify how clusters of homologous RNA structures identified by our method can be used to search for homologous structures across reference genomes and transcriptomes to generate a map of functionally related RNA structure motifs.

## Results

### Ensemble-guided pairwise RNA structure alignment

**Fig. 1:**
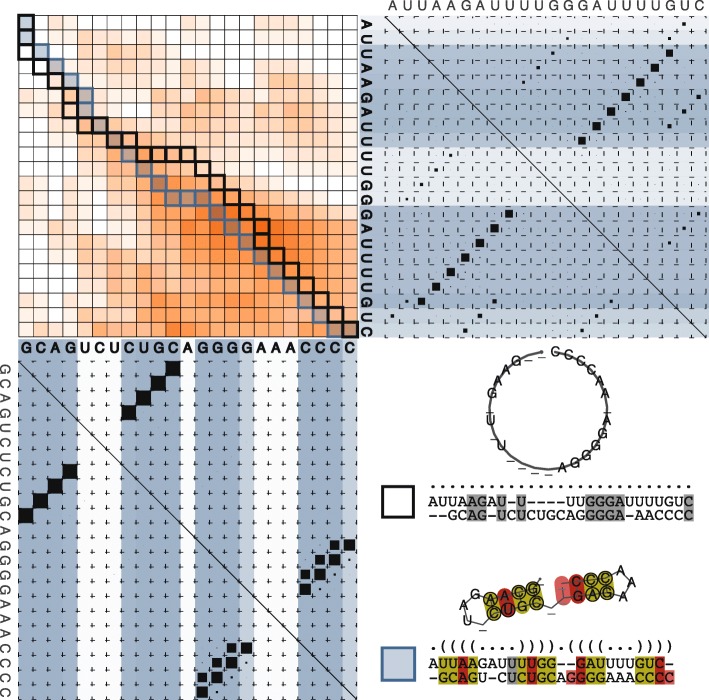
Schematic of a pairwise alignment with DotAligner. A dynamic programming matrix is first filled in based on the similarity in sequence and cumulative base-wise pairing probabilities of two RNA sequences (top left: colour intensity indicates cumulative similarity score). A partition function over all pairwise alignments is calculated and interrogated for structural compatibility by stochastic backtracking (not illustrated). Two ensembles over all secondary structures are considered for this purpose (bottom left and top right dot plots: blue lines indicate cumulative base-wise pairing probabilities). The final scoring uses the base pair probabilities in the dot plots. This effectively warps the optimal sequence alignment path (top left, black outline) towards one that includes structural features (top left, blue outline and fill). In the bottom right, the optimal sequence alignment and associated consensus secondary structure is contrasted to that produced by DotAligner, exposing the common structural features hidden in the suboptimal base pairing ensemble of both sequences

We developed an algorithm that leverages the diversity of suboptimal solutions from a partition function of RNA alignments to identify an optimal sequence-structure alignment of two RNAs. The algorithm, termed DotAligner, overcomes the limitations of comparing unique RNA secondary structures (such as minimum free energy predictions) to yield a pairwise alignment that considers mutual base pair probabilities. A schematic of how DotAligner functions is illustrated in Fig. 1.

DotAligner was developed with an emphasis on runtime performance to facilitate all-vs-all pairwise comparisons of RNA structures on large data sets. Consequently, it uses pre-calculated RNA dot plots to perform alignments. It also uses the observation that a significant subset of stochastic sequence alignments between two RNAs will overlap the correct structure-based alignment, even though the optimal sequence alignment deviates significantly from the structural alignment [34]. The algorithm combines an alignment-envelope heuristic with a fold-envelope heuristic, which impose constraints on suboptimal sequence alignments and pre-calculated base pair probabilities, respectively. The alignment procedure consists of two steps, each considering base pair probabilities: (1) generating a partition function of pairwise probabilistic string alignments and (2) stochastic sampling of string alignments and scoring of aligned dot plots. Existing building blocks are integrated to DotAligner in a novel way. A StrAL-like score is applied during the dynamic programming in step 1, then a CARNA-like score is used to score the aligned dot plots in step 2, and, lastly, the partition function in step 1 and sampling in step 2 are adapted from ProbA [34]. The detailed implementation and mathematical description of DotAligner can be found in Additional file 1.

### Evaluation of pairwise alignment quality

**Fig. 2:**
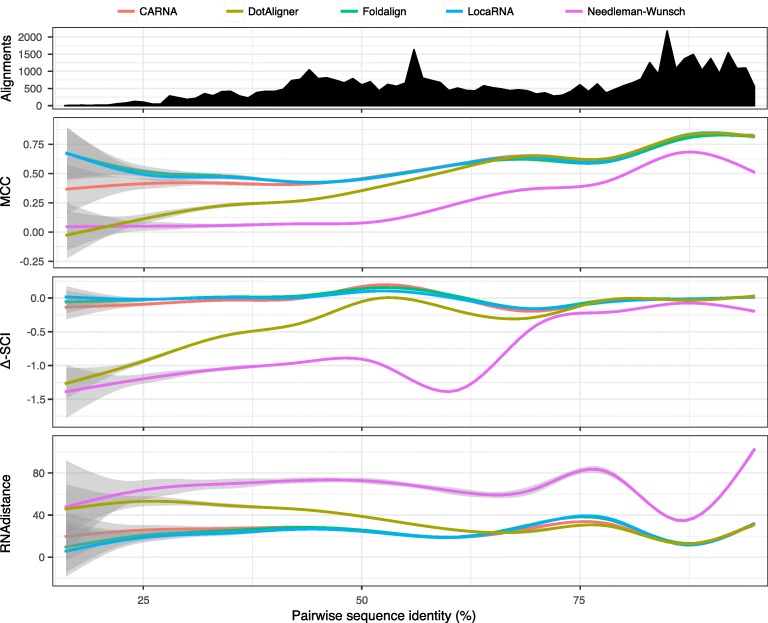
Comparison of RNA structure alignment quality as a function of sequence identity. BRAliBase 2.1 reference RNA structure alignments were submitted to five different pairwise alignment algorithms, including the Needleman–Wunsch sequence-only alignment algorithm. Top: The total number of surveyed alignments as a function of pairwise sequence identity. The Matthews correlation coefficient (MCC), the difference in the structural conservation index (*Δ*-SCI) and RNAdistance calculated topological edit distance between the RNAalifold consensus of the computed alignment and the reference BRAliBase 2.1 alignment consensus are compared in the lower three plots. MCC Matthews correlation coefficient, SCI structural conservation index

We first tested DotAligner on BRAliBase 2.1 pairwise RNA structure alignments, a reference data set specifically designed for algorithm benchmarking [19, 35] (see ‘Methods’). In this application, DotAligner seemingly performs worse than three other state-of-the-art algorithms, namely CARNA [30], FoldAlign [22, 36] and LocaRNA [25], as well as the Needleman–Wunsch pairwise sequence alignment algorithm, which ignores RNA structure content (Fig. 2). When comparing how well the algorithms perform as a function of the pairwise sequence identity of BRAliBase 2.1 reference alignments, DotAligner produces alignments of lesser quality than comparable RNA structure alignment tools, particularly below 60 % sequence identity, albeit with better accuracy than sequence-only alignments. Upon closer inspection, DotAligner outperforms the other tools around the 65–80 % sequence identity range. As mentioned in the next section, this roughly corresponds to the average pairwise intra-family sequence identity of Rfam clans.

Interestingly, many of the pairwise structure alignments produced structural conservation index (SCI) scores above those from the BRAliBase 2.1 reference alignments (Fig. 2). The SCI represents the alignment consensus energy normalised by the average energy of the single sequences folded independently [37]. It has been shown to be one of the most reliable metrics for conserved RNA structure detection [38]. With the exception of DotAligner, the other RNA structure alignment tools display, on average, SCI values above 0 in the 45–60 % identity range, suggesting that the underlying optimisation algorithms tend to overestimate the number of paired bases in consensus RNA structure predictions.

DotAligner’s capacity to produce competitive pairwise alignments is demonstrated via a 5S-adenosyl methionine riboswitch (Rfam clan RF00634, Additional file 2: Figure S1). In the Rfam alignment, the two representative sequences (AM420293_1 and CP000580_2_6) have a sequence identity of 55 %. Pure sequence alignment increases this to 69 %, but fails to align most structural features. DotAligner’s pairwise probabilistic string alignment (step 1) creates an alignment with pairwise sequence identity (PID) = 56 %, which is increased to PID = 63 % through DotAligner’s sampling. The number of correctly aligned suboptimal base pairs increases via DotAligner’s sampling. In this example, the alignment scores do not differ very much between DotAligner’s optimal string alignment (step 1) and the best sample (step 2) (0.58 and 0.60, respectively), despite a ∼25× increase of runtime through sampling (*s*=1000 in this example). As justified below, the benefits of sampling are outweighed by other properties of the algorithm.

### Fast and accurate classification of RNA structures

The intended application of DotAligner is the identification and clustering of RNA structural motifs from a large and diverse set of sequences of interest. Therefore, we evaluated the ability of DotAligner to distinguish between distinct structured RNA species from a heterogeneous sample of known RNA structure families. We performed all-vs-all pairwise structure alignments of stochastically sampled Rfam sequences, which were selected with constraints on their sequence composition (PID) to control for and ascertain any sequence-dependent biases (see ‘Methods’). DotAligner alignment scores were then compared to a binary classification matrix representing the distinct Rfam families (Additional file 2: Figure S3).

**Fig. 3:**
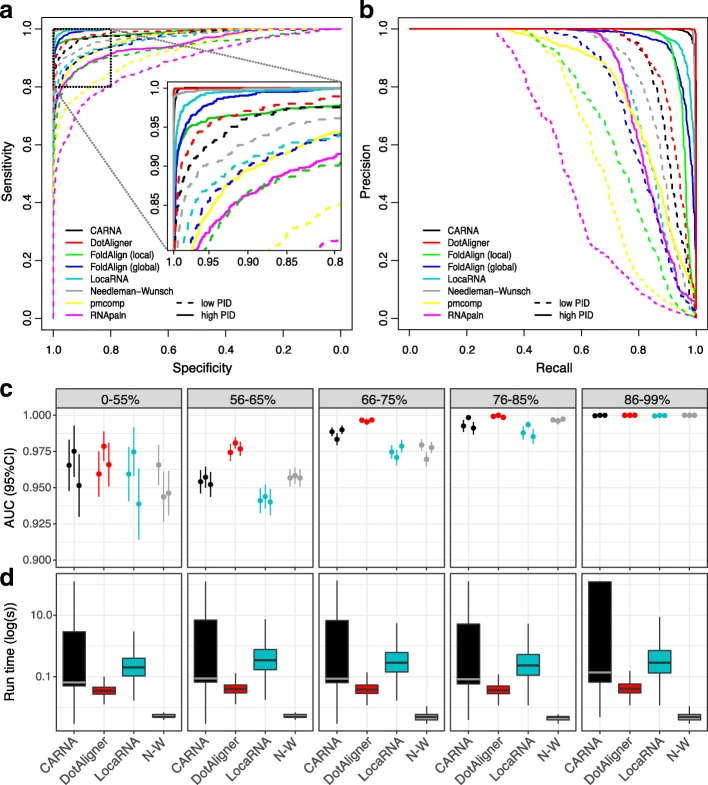
Classification of known RNA structures. **a** Receiving operator characteristic (ROC) curves measuring the classification accuracy of the surveyed algorithms by contrasting their computed similarity matrices to a binary classification matrix of Rfam sequences (1 if the sequences are in the same family or 0 if different). High PID = 56–95 % pairwise sequence identity from the provided Rfam alignment; low PID = 1–55 %. **b** Precision vs recall curve. **c** Area under the curve (AUC) of ROC values with 95 % confidence intervals for the top four performing algorithms across five ranges of pairwise sequence identity, as calculated from a variant of the Needleman–Wunsch algorithm with free end gaps. The three replicates correspond to stochastically sampled sequences from Rfam 12.3 (see Additional file 2: Table S1). **d** Runtime distribution of single-thread computation on a 2.6 GHz AMD Opteron processor (note, a fixed upper limit of 120 s was imposed for CARNA). AUC area under the curve

Despite the seemingly poor quality of pairwise alignments generated by DotAligner, it reproduces the known classification of Rfam structures more accurately, in general, than the other surveyed pairwise RNA structure alignment tools (Fig. 3 and Additional file 2: Table S1). In fact, only when the average pairwise sequence identity drops below 55 % for a given set of homologous RNA structures do the other algorithms perform comparably to DotAligner (Fig. 3 c). Interestingly, the sequence alignments produced by Needleman–Wunsch are able to cluster Rfam sequences into their respective clans well compared to more specialised RNA structure alignment tools, suggesting that most Rfam clans present sufficient stretches of local sequence identity to cluster them appropriately. Indeed, realigning the sequences from Rfam seed alignments based on their sequence alone, while permitting free end gaps to evaluate local sequence similarity, shifts the median pairwise sequence identity from 59 % to 72 % (Additional file 2: Figure S4).

The efficacy of the heuristics implemented in DotAligner is further accentuated by its runtime, which consistently lies between simple sequence alignment and more sophisticated RNA structure alignment algorithms (Fig. 3 d and Additional file 2: Figure S5). The impact of sequence length does not correlate with area under the curve (AUC) scores, but it increases the runtime in a polynomial way (Additional file 2: Figure S6).

### Density-based clustering of homologous RNA structures

Given DotAligner’s accurate clustering of known structured RNA using binary classification, we subjected its output to cluster analysis to identify and extract input sequences that display common sequence-structure motifs. Will et al. applied hierarchical clustering to the dissimilarity matrices produced by LocaRNA to organise sequences based on their structural homology [25]. However, this does not apply a cut-off that can be used to extract accurate novel clusters of structurally homologous sequences in an unsupervised manner. We attempted to achieve this by applying a statistical threshold derived from bootstrapping the underlying data using pvclust [39], but this generated clusters of variable size that often spanned across many disjoint families (data not shown).

We, therefore, opted for a density-based clustering strategy that, in theory, can decipher clusters of varying density (i.e. subsets of the data with significantly greater sequence-structure homology). The OPTICS (Ordering Points to Identify the Clustering Structure) algorithm [40] was chosen for this, as it has very few parameters to optimise. OPTICS is a derivative of the Density-Based Clustering for Application with Noise (DBSCAN) algorithm [41], which, as its name states, is suitable for noisy data, such as RNA immunoprecipitation followed by high-throughput sequencing. We benchmarked the two main OPTICS clustering parameters – the steepness threshold **xi** and the minimum number of points in a cluster (Additional file 2: Figure S7) – on a pooled set of 580 stochastically sampled Rfam sequences encompassing various ranges of sequence similarity, as well as a corresponding set of 580 dinucleotide shuffled controls (see ‘Methods’). After performing all-vs-all pairwise alignments with DotAligner, we evaluated the effect of OPTICS parameters on clustering performance, revealing that a minimum of four points (or sequences) and a steepness threshold of 0.006 gave the best results (Additional file 2: Figure S7A).

**Fig. 4:**
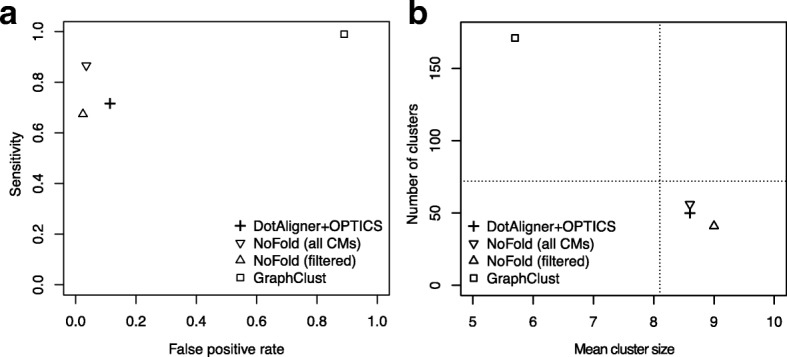
Comparative clustering benchmark of Rfam sequences and their shuffled controls. Clustering performance metrics of three algorithms on 580 reference Rfam structures and their dinucleotide-shuffled controls. **a** Sensitivity vs false positive rate. **b** Qualitative cluster statistics (the horizontal dashed line indicates the real number of clusters from unique Rfam families). CM covariance model

In comparison to GraphClust, the combination of DotAligner and OPTICS performs comparably well (Fig. 4, Table 1, Additional file 2: Table S2). The default version of NoFold nonetheless outshines DotAligner at clustering known Rfam families. However, it intrinsically employs Rfam covariance models (CMs) that are also present in the test data; therefore, this specific application is likely to be subject to over-fitting. We, thus, removed 72 CMs associated with the Rfam sequences in our benchmarking data set from the NoFold algorithm, which yielded lower sensitivity and less accurate qualitative cluster metrics than the DotAligner and OPTICS combination, while its specificity increased slightly despite removing CMs from its classification set.

**Table 1:** Comparative clustering performance

Algorithm	Number of	Sensitivity	Specificity	Accuracy
	clusters			
DotAligner+OPTICS	53	0.716	0.886	0.802
GraphClust	201	0.990	0.110	0.635
NoFold (all CMs)	62	0.866	0.965	0.916
NoFold (filtered)	45	0.674	0.976	0.826

### Identifying protein-binding RNA motifs from eCLIP data

**Fig. 5:**
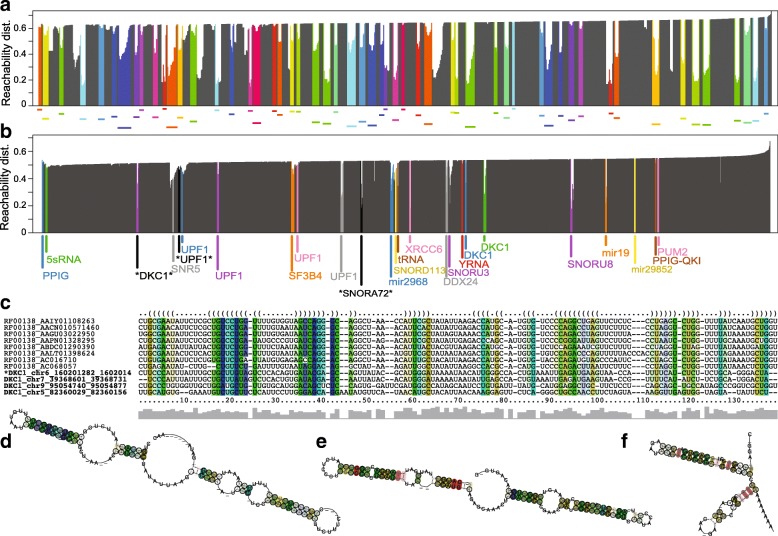
De novo homologous RNA motif identification. **a,b** Reachability plots of OPTICS clustering display the OPTICS-derived ordering of points (*x*-axis) and their distance to the nearest neighbour (*y*-axis). Colours represent significant clusters. **a** Clustering of Rfam benchmarking data indicating the distance to the nearest neighbour. **b** Clustering of 2650 ENCODE eCLIP peaks + 100 Rfam controls that overlap evolutionarily conserved secondary structure predictions. The dominant RNA binding protein in each cluster is displayed next to significant clusters. Those with an asterisk are portrayed below. **c** Multiple structure alignment generated by mLocaRNA on the sequences from a cluster containing both Rfam SNORNA72 sequences and DKC1 (a snoRNA-binding protein) eCLIP peaks. An unannotated DKC1-bound sequence is marked with an asterisk. **d–f** RNAalifold-predicted consensus RNA secondary structures: **d** Structure of the alignment displayed in (**c**). **e** Structure of a cluster of impartially detected DKC1-bound snoRNAs. **f** Structure of a novel UPF1-bound motif. dist. distance

The optimised parameters for OPTICS clustering of DotAligner output were incorporated into a high-performance computing pipeline that extracts clusters of homologous RNA structure motifs from a set of input sequences (see ‘Methods’). This pipeline was applied to eCLIP sequencing data from 44 RNA binding proteins from the ENCODE consortium [42], with 100 positive control sequences from Rfam (Additional file 2: Table S3). From 2650 high-confidence eCLIP peaks (>eightfold-enrichment vs background, *P* value <10^−4^) that overlap evolutionarily conserved secondary structure predictions, 25 significant clusters of homologous RNA were detected, including all 11 positive controls (Fig. 5).

Indeed, the *spike-in* Rfam sequences facilitate the identification of similar RNA structures, such as the homologues to SNORNA72 depicted in Fig. 5 c, d. The four additional sequences that co-cluster with SNORNA72 controls are all associated with the DKC1 protein, which binds to H/ACA snoRNAs. Furthermore, three of the DKC1-bound peaks are annotated as snoRNAs in the Gencode 24 reference, while the fourth is not annotated as a snoRNA despite strong sequence and structure similarity, highlighting how this method can successfully identify and cluster new RNA structure motifs. Another example is the Y RNA cluster, which contains three sequences homologous to this Rfam family that are also associated with the TROVE2 protein, which binds to misfolded non-coding RNAs, pre-5S rRNA and Y RNAs.

Our method also identifies RNA structure families impartially, as exemplified by several clusters of DKC1-associated sequences, which present consensus secondary structures indicative of snoRNAs (Fig. 5 e). Closer inspection of the corresponding eCLIP peaks revealed that these sequences are indeed annotated as snoRNAs in Gencode. There are also examples of de novo structural motifs that are associated with RNA-binding proteins with no previously known binding sites, such as an UPF1-dominated cluster (Fig. 5 f) composed of a structure motif belonging to ALU repeats (Additional file 2: Figure S8). When searching the human genome for homology to the RNA structure motif derived from this cluster, most ALU elements are detected, as well as a few other repeat-containing sequences. Interestingly, 998 homologues to the motif did not localise to ALU elements (Additional file 2: Figure S8C, D), 58 % of which overlap miTranscriptome reference transcripts [43].

## Discussion

The increasing accessibility of next-generation sequencing and immunoprecipitation protocols provides large resources for in-depth transcriptome and interactome profiling. Elucidating the structural features of RNAs associated with RNA-binding proteins and RNP complexes, combined with the systematic classification of their genome- or transcriptome-wide occurrence, can identify recurrent functional motifs that may form components of regulatory networks. Pragmatically, the method we describe facilitates this process by enabling rapid and unsupervised clustering of RNA structure motifs with reasonable accuracy. We also show that clustering eCLIP sequences can identify new RNA structures and their homologues throughout the genome (Additional file 2: Figure S8A–C), which can be used to assign putative functions to non-coding loci and categorise them accordingly.

Given its relative speed and accuracy, DotAligner can be used to generate larger (dis)similarity matrices for cluster analysis than other pairwise structure alignment algorithms, or at least produce them with reasonable computational power. In addition to its speed, the strength of DotAligner lies in its capacity to accurately score structurally homologous RNA sequences and the suboptimal structural landscape of RNAs, reducing several dimensions of information into a single discriminative numeric value. Our results show that this can be sufficient to extract structurally and functionally related sequences from a large amount of noisy input. It is an ideal application for screening high-throughput sequencing data – such as RNA immunoprecipitation data – for common structural motifs.

The algorithm generates pairwise alignments that differ qualitatively to reference structural alignments at lower ranges of sequence identity, but it performs better than more complex algorithms within ranges of sequence similarity that substantially overlap those of functionally related RNAs, as presented in Rfam. This could be a consequence of refining the runtime parameters through testing on independently and stochastically sampled Rfam sequences. It is not impossible that other algorithms could undergo comparable parameter optimisation. However, the significantly higher computational complexity of other related tools compared to our method makes it fairly difficult (and resource intensive) to perform such brute-force parameter optimisation.

High-throughput CLIPseq data pose a challenge for the detection of consensus motifs since several molecules that are in close physical proximity to the target molecule can co-precipitate together. Consequently, other RNA sequences that do not directly bind to the target protein may be present. We have shown that our method is, nonetheless, suitable for such noisy biological data. For example, the UPF1 cluster we describe may be an example of an indirect binding event, as UPF1 directly interacts with STAU1, a double-stranded RNA-binding protein that has been reported to target ALU sequences [44]. Other clusters identified in our eCLIP analysis have sequences from more than one target protein clustered together, which raises the possibility that a common RNA structure motif may be bound by different proteins, either as part of a quaternary complex or as a common, competing binding target. We privilege this hypothesis over one of spurious false-positive clustering given our benchmark results and the observation that very few clusters were observed when analysing less stringently filtered eCLIP peaks (data not shown).

DotAligner has several variables that can influence the clustering results and speed depending on the type of input data. The most influential variables are the weight between sequence and structure similarity, and the exploration depth of suboptimal alignments in the stochastic backtracking. We have shown that stochastic sampling of suboptimal string alignments improves the alignment of RNA dot plots. However, the performance increase does not outweigh the increase in runtime associated with sampling suboptimal sequence alignments. Our Rfam clustering benchmark using a binary classification strategy has shown that the best trade-off between alignment accuracy and speed comes with the abandonment of sampling, as supported by the de novo structures identified from the ENCODE eCLIP data. Future optimisation of DotAligner parameters will likely increase its usability. For example, dynamic parameters could be implemented that adjust the degree of sampling diversity and number of samples based on the sequence identity obtained from step 1 of DotAligner. This could tune the algorithm’s performance based on the nature of the input, potentially improving DotAligner’s performance across all ranges of sequence identity. Another potential enhancement could be achieved in the stochastic sampling by considering only elements of the ensemble with probabilities larger than a threshold. By doing so, we could (1) reduce the number of useless samples, (2) guarantee that cells of high probability are passed (suboptimal structures) and (3) leave time/samples to explore the ensemble space (slightly modified alignments by limiting sample diversity) around these suboptimals.

Another great challenge lies in the accurate depiction of RNA structure motif boundaries. Whereas global structures may stabilise the RNA molecule, local structural domains are often sufficient for recognition by RNA binding proteins. A strategy to find optimal local alignments would be desirable for this. DotAligner can search for semi-local alignments by introducing penalty-free gaps at the sequence extremities (note, LocaRNA also supports this functionality). In this study, we did not investigate the optimisation of these local pairwise similarity scores, because they may miss parts of the functional units (RNA structure) and, hence, hinder the search for optimal clusters. Instead, we circumvented this issue by overlapping eCLIP peaks to evolutionarily conserved RNA secondary structure predictions with well-characterised flanking helices supported by base pair covariation [6]. While preparing this manuscript, a complementary and comprehensive data set of evolutionarily conserved RNA secondary structures was published [45]. Its application could further increase the number of eCLIP peaks with accurate structural motif boundaries. Alternatively, RNA structure boundaries can be refined by, for example, using alternative strategies, such as computational boundary refinement with LocaRNA-P [46] or improving the biological data with enzymatic probing with the double-stranded RNase T1 endoribonuclease.

## Conclusion

An efficient pairwise RNA sequence alignment heuristic, which intrinsically considers suboptimal base pairings, accurately discriminates between distinct structured RNA families. When combined with a noise-tolerant density-based clustering algorithm, this approach identifies known and novel RNA structure motifs from a set of input sequences without a priori knowledge. The resulting RNA structure motifs are subsequently used to identify homologues in the human genome, improving the annotation of lncRNAs and increasing the repertoire of functional genetic elements.

## Methods

### Benchmarking and parameter optimisation

The DotAligner algorithm implements several parameters that first need to be tuned before it can be applied to biological sequence analysis. All combinations of core parameters were tested on the 8976 pairwise RNA structure alignments curated in the BRAliBase 2.1 reference data set [35]. We first tested all combinations of the following parameters: ***k*** and ***t*** from 0 to 1 in increments of 0.1; ***o*** and ***e*** from 0.2 to 1 in increments of 0.2. For each set of parameter combinations, the number of alignments producing identical structural topologies to the reference alignment was determined using RNAdistance, SCI, a robust measure of RNA structural alignment integrity [38] based on the minimum free energy (MFE), and the Matthews correlation coefficient (MCC) of *predicted* and *reference* RNA secondary structure were also calculated for all resulting alignments:

$$\begin{aligned} \text{MCC} &= \frac{\left(\text{TP} \times \text{TN}\right) - \left(\text{FP} \times \text{FN}\right)}{ \sqrt{\left(\text{TP} + \text{FP}\right)\left(\text{TP} + \text{FN}\right)\left(\text{TN} + \text{FP}\right)\left(\text{TN} + \text{FN}\right) }},\\ \Delta \text{SCI} &= \text{SCI}_{\text{predicted}} / \text{SCI}_{\text{reference}}. \end{aligned} $$

Here, true positives (TP) is the number of representatives from the dominant Rfam family in a cluster. False positives (FP) is the number of non-dominant Rfam family representatives in a cluster, or the number of clusters where there is no dominant Rfam family (i.e. equally represented families), or the number of clusters where the dominant sequence is a negative control. False negatives (FN) is the number of Rfam sequences that fail to cluster. True negatives (TN) is the number of negative control sequences that fail to cluster. Moreover, $\text {SCI} = \text {MFE}_{\text {consensus}} / \overline {\text {MFE}}_{\text {single}}$.

The baseline parameters were then selected via a product rank of these two metrics and subjected to refinement using a binary classification approach, described in the next section.

### Binary classification of RNA secondary structure families

Further refinement of the optimal parameters was performed using a binary classifier for two sets of 200 stochastically sampled Rfam entries with published structures: (i) a low pairwise sequence identity (PSI) set and (ii) a high PSI set, where any two sequences from the same family share between 0–55 % and 56–95 % PSI, respectively (Fig. 3 a, b). The Java implementation of this algorithm, derived from [6], can be found in Additional file 1. Further investigation of the impact of local sequence similarity on algorithmic performance was done by sampling all seed alignments of Rfam version 12.3 via three replicates of our stochastic sampling procedure. The sequences were then stripped of gaps and pseudo-knots (not present in the preliminary Rfam version 12.0 alignments), and realigned with a variant of Needleman–Wunsch enabling free end gaps. The samplings were limited to five ranges of sequence identity, as presented in Fig. 3 c.

A binary classification matrix was then constructed, where sequences *x* and *y* have a score of 1 if they belong to the same Rfam family, or a score of 0 if they do not. The similarity matrix resulting from all-vs-all pairwise comparisons with DotAligner was tested for accuracy using the AUC of the receiving operator characteristic, as calculated by the R package pROC [47]. A more restricted range of parameter values was then tested on both data sets. A ranked sum for both data sets of the AUC was performed to determine the default runtime parameters for DotAligner, namely *θ*=0.5, *κ*=0.3, *g*_o_=1 and *g*_ext_=0.05 (Additional file 2: Table S4). Parameter *θ* (or -t in the command line) is the weight of sequence similarity compared to the similarity of unpaired probabilities, *κ* (or -k) is the weight between sequence-based similarity and dot plot similarity, *g*_o_ (or -go) is the gap opening penalty and *g*_ext_ (-go) is the gap extension penalty. Sampling-specific parameters *s* (number of samples) and *T* (measure of sampling diversity) had minimal impact in the refined parameter optimisation from sampled Rfam clans, although the parameters can increase alignment scores in low and medium pairwise sequence identity ranges (Additional file 2: Figures S1 and S2A). We also show that, on average, the alignment score saturates after 1000 samples of the stochastic backtracking for *T*=0.25 (Additional file 2: Figure S2B). CARNA version 1.2.5 was run with parameters --write-structure --noLP --time-limit=120000. LocaRNA version 1.7.13 was run with parameter --noLP. FoldAlign version 2.1.1 was run with and without parameter -global. Default parameters were used for pmcomp, downloaded from https://www.tbi.univie.ac.at/RNA/PMcomp/, and RNApaln version 2.3.5. The custom implementation of Needleman–Wunsch can be found in the GitHub repository associated with this work, as can the benchmark implementation scripts.

### Clustering RNA structures with randomised controls

OPTICS benchmarking was performed by stochastically sampling the collection of Rfam 12.0 seed alignments using the Java program GenerateRfamsubsets.java (see Additional file 1) with three ranges of pairwise sequence identity: 1–55 %, 56–75 % and 75–95 %, a minimum of five representative sequences per family, and sizes ranging between 70 and 170 nt. The resulting 580 unique sequences were then shuffled while controlling their dinucleotide content with the easel program included in the Infernal (v1.1.2) software package [48] with option -k 2. The 1160 sequences were submitted to all-vs-all pairwise comparisons with DotAligner and the scores were inverted and normalised (min=1, max=0) into a dissimilarity matrix, which was then imported into the R statistical programming language, converted into a ‘dist’ object without transformation, and subjected to OPTICS clustering as implemented in the dbscan CRAN repository with a range of parameters (see Fig. 4 a, b).

Other tested RNA clustering approaches were GraphClust and NoFold. We ran GraphClust version 0.7.6 inside the Docker image provided with RNAscClust with default parameters. NoFold version 1.0.1 uses 1973 Rfam CMs by default as empirical feature space. For the NoFold (all CMs) variant, we ran the program with default parameters, whereas for the NoFold (filtered) variant, we reduced the feature space to 1902 CMs by removing Rfam families from our benchmark set.

The following clustering performance metrics were used:

$$\begin{aligned} \text{Sensitivity (recall)} &= \text{TP} / (\text{TP} + \text{FN}), \\ \text{Specificity} &= \text{TN} /(\text{TN} + \text{FP}), \\ \text{False positive rate} &= 1 - \text{Specificity}, \\ \text{Precision} &= \text{TP} / (\text{TP} + \text{FP}), \\ \text{Accuracy} &= (\text{TP} + \text{TN}) / (\text{TP} + \text{TN} + \text{FP} + \text{FN}). \end{aligned} $$

### Clustering of protein-bound evolutionarily conserved RNAseq reads

The genomic coordinates of ENCODE eCLIP peaks were downloaded in bed format from the April 2016 release via the ENCODE portal (https://www.encodeproject.org/search). The resulting 5,040,096 peaks were filtered to keep only those with ≥eightfold enrichment over the total input background and an associated *P* value ≤10^−4^. Furthermore, peaks were merged if they overlapped by more than 50 nt to avoid over-representing the same sequence (Additional file 1). The remaining peaks were subsequently filtered by retaining only those that have a same-strand overlap with any evolutionarily conserved structure predictions from [6]. Finally, the associated genomic sequences were extracted into a fasta file, which was supplemented with 100 reference RNA structures from 11 Rfam families (see Additional file 2: Table S3). Merging, overlap and sequence extraction operations were performed with Bedtools version v2.26.0.

The normalised similarity matrix resulting from all-vs-all pairwise comparisons with DotAligner was then subjected to clustering with the dbscan 1.1-1 R package from Michael Hahsler (https://github.com/mhahsler/dbscan) using the command opticsXi(optics(D, eps=1, minPts=4, search=~dist~), xi = 0.006, minimum=T). The sequences for each cluster were then extracted and submitted to multiple structure alignment with mLocaRNA version 1.9.1 using parameters --probabilistic --iterations=10 --consistency-transformation --noLP.

## Additional files

Additional file 1 Supplementary methods describing the DotAligner implementation in detail, RNA structure clustering and eCLIP data processing methodologies. (PDF 602 kb)

Additional file 2 Supplementary tables and figures with descriptions. (PDF 3890 kb)
