# Supplementary material for: DotAligner: identification and clustering of RNA structure motifs
Source: Genome Biol. 2017 Dec 28;18:244. doi: 10.1186/s13059-017-1371-3 (PMC5747123; doi:10.1186/s13059-017-1371-3)
Supplement: Supplementary file 1 — Supplementary methods describing the DotAligner implementation in detail, RNA structure clustering and eCLIP data processing methodologies. (PDF 602 kb) [file 13059_2017_1371_MOESM1_ESM.pdf]

Additional file 1 — Supplementary methods

The pipelines, scripts and some programs used in this work can be found in the GitHub repository associated to this work at `https://github.com/noncodo/BigRedButton`.

1. `DotAligner` implementation

The weight W of alignment A of two arc-annotated sequences (S_a, P_a) and (S_b, P_b) has been defined by [29] as

$$\begin{aligned} W(A) &= \sigma(A) + \tau(A) + \gamma(A) \\ &= \sum_{(i,i')\in A} \sigma(i,i') + \sum_{\substack{(i,j)\in P_a, \\ (i',j')\in P_b, \\ (i,i')\in A, \\ (j,j')\in A}} \tau(i,j,i',j') + \gamma \times N \end{aligned} \tag{1}$$

where S is a sequence and P is a base pair probability matrix, $\sigma(i,i')$ is the similarity of sequence positions $S_a[i]$ and $S_b[i']$, $\tau(i,j,i',j')$ is the similarity of arcs $(i,j) \in P_a$ and $(i',j') \in P_b$, and γ is the gap cost associated with each sequence position that is not matched ($N = |S_a| + |S_b| - 2|A|$). The alignment problem finds the maximal $W(A)$. As its solution is MAX-SNP-hard, in practise heuristics are used to find near-optimal solutions.

`DotAligner` solves the related problem of aligning two base pair probability matrices (*dot plots*). A major criteria for the implementation was a fast running time enable all-vs-all pairwise structural alignments and the associated distance (dissimilarity) matrix, which can be used for cluster analysis of large data sets [25]. Consequently, it employs a heuristic alignment-envelope, which imposes constraints to sub-optimal string alignments, and a fold-envelope, which imposes constraints to pre-calculated base pair probabilities, to build pairwise sequence-structure alignments.

Below, we describe the alignment procedure and weight functions. The alignment procedure consists of two steps:

1. Partition function of pairwise probabilistic string alignments;
2. Stochastic sampling of string alignments and scoring of aligned dot plots.

1.1 Partition function of pairwise probabilistic string alignments

In step 1 the computation of the partition function $Z(T)$ over all canonical pairwise string alignments A is adapted from probA [34]:

$$Z(T) = \sum_{A} exp(\beta W(A)), \tag{2}$$

where $\beta = 1/T$. Then the probability of a specific alignment A is defined as:

$$Pr(A;T) = \frac{1}{Z(T)} exp(\frac{W(A)}{T}), \tag{3}$$

The parameter T is analogous to the temperature in the thermodynamic interpretation of the alignment problem and determines the relative importance of the optimal string alignment. If $T = 1$ then we recover the 'true' probability, if $T \to 0$ then $Pr(A;0) = 0$ for all alignments with a score $W(A)$ less then the score of the optimal string alignment, and if $T \to \infty$ then all alignments have the same $Pr(A,\infty) = 1/Z(\infty)$. Hence, T controls the search space of suboptimal alignments for step 2. The algorithm uses the dynamic programming algorithm of Gotoh [50] which has running time of $O(N^2)$. The weight function $W(A)$ of the probA implementation is changed to explore the ensemble of dot plot alignments. We reduce the sequence-structure alignment problem to a two-dimensional problem similar to the metric introduced in StrAL [28]. Hence, step 1 considers only the similarity σ and the gap cost γ described in equation 1:

$$W_{\text{Step1}}(A) = \sigma(A) + \gamma(A) \tag{4}$$

The similarity $\sigma(i,i')$ for matched sequence positions $S_a[i]$ and $S_b[i']$ takes into account sequence similarity M_{Seq} and the similarity in their unpaired probabilities $\Delta\omega(i,i')$ weighted by the parameter θ:

$$\sigma(i,i') = \theta \times M_{Seq}^{(i,i')} + (1-\theta) \times \Delta\omega(i,i') \tag{5}$$

$M_{Seq}^{(i,i')}$ is 1 if sequence positions $S_a[i]$ and $S_b[i']$ match and else 0. The similarity of unpaired probabilities is defined as

$$\Delta\omega(i,i') \in \begin{cases} 0 & \text{if } \omega(i) == 0 \\ & \text{and } \omega(i') == 0 \\ 1 - |\omega(i) - \omega(i')| & \text{else} \end{cases} \tag{6}$$

so that $\Delta\omega = (0,1)$.

The gap term in equation 1 is replaced with affine gap costs:

$$\gamma(A) = l \times g_o + (N - l) \times g_{ext} \tag{7}$$

where l is the number of initiation gaps, N is the number of all gaps, g_o is the penalty for opening a gap and g_{ext} is the penalty for gap extensions. Start and end gaps can be considered as free (set parameter `--free-endgaps`).

1.2 Stochastic sampling of string alignments and scoring of aligned dot plots

Here, a properly weighted sample of stochastic pairwise string alignments in the alignment ensemble is examined across both sequences for sequence-structure similarity. The

stochastic backtracking is adapted from probA [34] for selecting s suboptimal string alignments A_s. The combined score (weight) W_{Step2} is a variant of equation 1 to explore the similarity of the corresponding dot plot alignments:

$$W_{\text{Step2}}(A_s) = \kappa \times \frac{W_{\text{Step1}}(A_s)}{|A_s|} + (1-\kappa) \times \frac{\tau(A_s)}{|\text{Match}_{A_s}|^2} \tag{8}$$

where the parameter κ weights for each alignment A_s between the sequence-based similarity $W_{\text{Step1}}(A_s)$ normalised by alignment length $|A_s|$ and dot plot similarity $\tau(A_s)$ normalised by the number of aligned bases $|\text{Match}_{A_s}|$ in alignment A_s. Similar to equation 5 the dot plot similarity τ sums the parameter θ weighted similarity of aligned base pairs M_{paired} and the similarity in their pairing probabilities $\Delta\psi$:

$$\tau(i,j,i',j') = \theta \times M_{paired}^{(i,j,i',j')} + (1-\theta) \times \Delta\psi(i,j,i',j') \tag{9}$$

where $M_{paired}^{(i,j,i',j')}$ is 1 if $S_a[i]$ and $S_a[j]$ as well as $S_b[i']$ and $S_b[j']$ form canonical base pairs (G-C, C-G, A-U, U-A, G-U or U-G) and else 0. The similarity in pairing probabilities $\Delta\psi$ is then calculated by

$$\Delta\psi(i,j,i',j') \in \begin{cases} 0 \text{ if } \psi(i,j) == 0 \text{ and } \psi(i',j') == 0 \\ 1 - |\psi(i,j) - \psi(i',j')| \text{ else} \end{cases} \tag{10}$$

For both sequences S_a and S_b, the pairing probability matrices P_a and P_b are computed in advance using McCaskill's algorithm, implemented in `RNAfold` or `RNAplfold`. The robustness of the alignment is improved by applying log-odds scores ψ of having a specific base pairing against the null model of a random pairing [25]:

$$\psi(i,j) = max\left(0, log\frac{P(i,j)}{p_0} / log\frac{1}{p_0}\right) \tag{11}$$

where p_0 is the expected probability for a pairing to occur at random. The term $log\frac{1}{p_0}$ is a normalization factor that transforms the scores to a maximum of 1. $P == 1$ results in $\psi = 1$, $P > p_0$ results in $\psi > 0$, and $P \leq p_0$ results in $\psi = 0$. This transformation gives weaker similarities if low base pair probabilities are compared, but stronger similarities for high base pair probabilities. Unpaired probabilities are handled in a similar way by

$$\omega(i) = max\left(0, log\frac{1-\sum_k P(i,k)}{p_0} / log\frac{1}{p_0}\right) \tag{12}$$

where p_0 is the expected probability for an unpaired base to occur at random.

1.3 Alternative model using substitution rates

Alternatively, the sequence and base pair similarities M_{Seq} and M_{paired} in equations 5 and 9 can be replaced by the statistical substitution models R_{Seq} and R_{paired}, respectively. In this (non-default) model of `DotAligner` R_{Seq} is multiplied with the ζ weighted sum of the similarity of unpaired probabilities $\Delta\omega$ and the similarity of upstream pairing probabilities $\Delta\omega^{up}$ (set parameter `--mutation-rates`):

$$\sigma(i,i') = R_{Seq}^{(i,i')} \times \zeta \times \Delta\omega(i,i') + R_{Seq}^{(i,i')} \times (1-\zeta) \times \Delta\omega^{up}(i,i') \tag{13}$$

R_{Seq} is a 4×4 matrix of probabilities for observing a given substitution relative to background nucleotide frequencies. We use the log-odd scores L from the RIBOSUM85-60 matrix introduced in [51] which are transformed to probabilities R_{Seq} by $2^{L(i,i')}/(1+2^{L(i,i')})$. The ratio of upstream pairing probability ω^{up} is defined as

$$\omega^{up}(i) = \sum_{k=1}^{i-1} \psi(k,i) / \sum_{k=1}^{|S|} \psi(k,i) \tag{14}$$

where $i \in S$, $|S|$ is the length of sequence S, and $\psi(k,i)$ is the pairing probability of sequence positions $S[k]$ and $S[i]$. The downstream pairing probability is implicitly considered in the weight function through the usage of unpaired probability and upstream pairing probability. The base pair similarity matrix M_{paired} can be replaced by a statistical substitution model R_{paired} which describes the probability for observing a given base pair substitution relative to background nucleotide frequencies:

$$\tau(i,j,i',j') = R_{paired}^{(i,j,i',j')} \times \Delta\psi(i,j,i',j') \tag{15}$$

The log-odd scores L from the RIBOSUM85-60 matrix [51] are transformed to probabilities R_{paired} by $2^{L(i,j,i',j')}/(1+2^{L(i,j,i',j')})$.

2. Clustering RNA structures with randomised controls

Below is the code used to calculate the accuracy and other performance metrics of the clustering benchmark of stochastically sampled RFAM entries. All files can be found on the associated `GitHub` repository `https://github.com/noncodo/BigRedButton`.

```
## R code -- R version 3.4.1
cat("File name","TP","TN","FP","FN","SENS","SPEC","ACC","\n",sep="\t",
    file="accuracies.tsv")
file.names <- dir(pattern="*_clust.tsv$")
for(x in 1:length(file.names)){
gc <- read.delim(file.names[x], header=F)
# for 1 - max V2
TP=0
FP=0
NumClust <- max(gc$V2)
for ( cl in 0:NumClust) {
```

```
  if ( cl %in% gc$V2 ) {
  v <- as.vector( gc$V1[ gc$V2 == cl ] )
  t <- sort( table( v ), decreasing=T )
  best <- as.integer( t[1] )
  cID <- names( t[ 1 ] )
  if ( cl == 0 ) {
    if ( cID == "shuffled" ) {
      FN <- length(v)-best
      TN <- best
    }
    else
      cat("Houston, we have a TN problem")
  }
  else {
    if ( cID == "shuffled" ) {
      FP = FP + length(v)
    }
    if ( is.na( as.integer( t[2] )) || as.integer( t[2] ) < best ) {
      TP = TP + best
      FP = FP + length(v)-best
    }
    else if ( as.integer( t[2] ) == best ) {
      # treat both as false positives
      FP = FP + length(v)
    }
  }
}}
TP
TN
FP
FN
SENS=TP / (TP + FN )
SENS
SPEC=TN / ( TN + FP )
SPEC
ACC=(TP + TN) / ( TP + TN + FP + FN )
ACC
cat(file.names[x],TP,TN,FP,FN,SENS,SPEC,ACC,"\n",sep="\t",
    file="accuracies.tsv", append=T)
}
```

3. eCLIP data processing

Data in .bigBed format was acquired from the ENCODE data hub from the following link: https://www.encodeproject.org/search/?type=Experiment&assay_term_name=eCLIP&files.file_type=bigBed+narrowPeak&month_released=April%2C+2016

```
#!/bin/bash

# Convert accessions to protein IDs
cut -f 1,16,29 metadata.tsv | sed 's/-human /_/g' | while read line
do
  F1=$(echo $line | awk '{print $1".bed"}' )
  F2=$( echo $line | awk '{ print $2".bed"}')
  cp $F1 $F2
done

# Rename files accordingly
```

```
for file in *bed
do
  mv $file $(head -n 1 $file | cut -f 4).bed
done

# Filter for greater than or equal to 8x fold enrichment
# And -log10( P-value ) greater than or equal to 4
for file in *rep0?.bed
do
  awk '{if ($7 >= 4 && $8 >= 4) print }' $file > ../filtering/${file}_filt3
done

#Intersect both replicates (>1 overlap)
for file in *rep01.bed_filt3 ; do
    >&2 echo "Processing "$file
    bedtools intersect -s -u -f 0.5 -a <( cut -f 1-6 $file ) -b <( cut -f 1-6
        ${file//rep01/rep02} ) > ${file}_1
    bedtools intersect -s -u -f 0.5 -b <( cut -f 1-6 $file ) -a <( cut -f 1-6
        ${file//rep01/rep02} ) > ${file}_2

    # merge peaks if they are close together
    bedtools merge -d 50 -s -delim "|" -c 4,5,6 -o first,count,first -i <( cat
        ${file}_1 ${file}_2 | sort -k 1,1 -k 2,2n ) >
        ${file%*.bed_filt3}_filt_0.5_merged_50_s.bed

   # intersect with ECS (in file ECS_congruous_sorted.bed6)
    bedtools intersect -wo -s -b ${file%*.bed_filt3}_filt_0.5_merged_50_s.bed
        -a ECS_congruous_sorted.bed6 >
        ${file%*.bed_filt3}_filt_0.5_merged_50_s_anyECS.bed
done

#merge all files into one
cat *_50_s_anyECS_merged.bed > All_ECS_merged_50nt_peaks.bed
# wc -l All_ECS_merged_50nt_peaks.bed
## 2650

#edit sequence names and get sequence from reference genome (hg19)
awk 'OFS="\t"{print $1,$2,$3,$4"_"$1"_"$2"_"$3"_"$6,$5,$6}'
     ./All_ECS_merged_50nt_peaks.bed > ./All_ECS_merged_50nt_peaks_renamed.bed
bedtools getfasta -s -name -fi ~/data/fasta/hg19.fa -bed
     ./All_ECS_merged_50nt_peaks_renamed.bed -fo
     ./All_ECS_merged_50nt_peaks_renamed.fasta

#combine with known control RNA structure
cat All_ECS_merged_50nt_peaks_renamed.fasta spike-ins.fasta >
     All_ECS_merged_50nt_peaks_renamed_spikeIns.fasta
```
