# Supplementary material for: DotAligner: identification and clustering of RNA structure motifs
Source: Genome Biol. 2017 Dec 28;18:244. doi: 10.1186/s13059-017-1371-3 (PMC5747123; doi:10.1186/s13059-017-1371-3)
Supplement: Supplementary file 2 — Supplementary tables and figures with descriptions. (PDF 3890 kb) [file 13059_2017_1371_MOESM2_ESM.pdf]

Additional file 2 — Supplementary Tables and Figures

Table S1. Uniqueness and diversity of stochastically sampled RFAM subsets

			# RFAM families		
Pairwise identity range	# sequences	% unique	Rep. 1	Rep. 2	Rep. 3
0-55	178	94.4	33	19	32
56-65	900	92.2	113	108	110
66-75	899	92.6	83	76	74
76-85	900	91.7	80	82	79
86-99	900	93.6	58	47	59

Table S2. List of RFAM families from benchmark that did not cluster

Sequence count	RFAM ID	RFAM family
2	RF00005	tRNA
5	RF00015	U4 spliceosomal RNA
8	RF00020	U5 spliceosomal RNA
5	RF00021	Spot 42 RNA
1	RF00026	U6 spliceosomal RNA
10	RF00059	TPP riboswitch (THI element)
5	RF00167	Purine riboswitch
11	RF00169	Bacterial small signal recognition particle RNA
13	RF00199	SL2 RNA
4	RF00374	Gammaretrovirus core encapsidation signal
11	RF00378	Qrr RNA
6	RF00386	Enterovirus 5' cloverleaf cis-acting replication element
6	RF00389	Bamboo mosaic virus satellite RNA cis-regulatory element
4	RF00444	PrrF RNA
17	RF00494	Small nucleolar RNA U2-19
2	RF00515	PyrR binding site
4	RF00550	Hepatitis E virus cis-reactive element
7	RF01685	6S-Flavo RNA
7	RF01697	Chlorobi-RRM RNA
6	RF01705	Flavo-1 RNA
4	RF01725	SAM-I/IV variant riboswitch
2	RF01728	STAXI RNA
7	RF01734	crcB RNA
1	RF01750	pfl RNA
6	RF01754	radC RNA
4	RF01764	yjdF RNA
5	RF02033	HNH endonuclease-associated RNA and ORF (HEARO) RNA

Table S3. List of control RNA structures

Sequences	RNA family	RFAM ID
5	5SRNA	RF00002
8	SNORA72	RF00138
10	SNORD113	RF00181
10	SNORU3	RF00012
10	SNORU8	RF00096
8	SNR5	RF01252
9	YRNA	RF00019
10	mir19	RF00245
7	mir2968	RF02093
6	mir29852	RF02095
17	tRNA	RF00005

Table S4. Rank-product of best `DotAligner` parameters

Parameters	low_PI rank	high_PI rank	rank product	low_PI AUC	high_PI AUC	AUC sum	Combined Time
k=0.3 t=0.5 o=1 e=0.05	1	112	112	0.983297903	0.996178994	1.97948	0.140273
k=0.3 t=0.8 o=1 e=0.05	181	1	181	0.959342489	0.997188985	1.95653	0.133496
k=0.3 t=0.5 o=1 e=0.05	2	110	220	0.983297903	0.996178994	1.97948	0.135262
k=0.3 t=0.5 o=1 e=0.05	3	109	327	0.983297903	0.996178994	1.97948	0.134188
k=0.3 t=0.8 o=1 e=0.05	184	2	368	0.959342489	0.997188985	1.95653	0.144565
k=0.3 t=0.5 o=1 e=0.05	4	113	452	0.983297903	0.996178994	1.97948	0.150288
k=0.3 t=0.8 o=1 e=0.05	182	3	546	0.959342489	0.997188985	1.95653	0.142137
k=0.3 t=0.5 o=1 e=0.05	5	114	570	0.983297903	0.996178994	1.97948	0.156738
k=0.3 t=0.5 o=1 e=0.05	6	111	666	0.983297903	0.996178994	1.97948	0.155101
k=0.3 t=0.8 o=1 e=0.05	185	4	740	0.959342489	0.997188985	1.95653	0.146729
k=0.3 t=0.5 o=1 e=0.05	7	115	805	0.983297903	0.996178994	1.97948	0.186257
k=0.3 t=0.5 o=1 e=0.05	8	116	928	0.983297903	0.996178994	1.97948	0.192388
k=0.3 t=0.8 o=1 e=0.05	186	5	930	0.959342489	0.997188985	1.95653	0.154183
k=0.3 t=0.5 o=1 e=0.05	9	117	1053	0.983297903	0.996178994	1.97948	0.210514
k=0.3 t=0.8 o=1 e=0.05	183	6	1098	0.959342489	0.997188985	1.95653	0.154234
k=0.3 t=0.5 o=1 e=0.05	10	119	1190	0.983297903	0.996178994	1.97948	0.285647
k=0.4 t=0.6 o=1 e=0.05	13	97	1261	0.983273039	0.996343919	1.97962	0.133738
k=0.3 t=0.5 o=1 e=0.05	11	118	1298	0.983297903	0.996178994	1.97948	0.269801
k=0.3 t=0.8 o=1 e=0.05	187	7	1309	0.959342489	0.997188985	1.95653	0.187293
k=0.4 t=0.6 o=1 e=0.05	14	101	1414	0.983273039	0.996343919	1.97962	0.144514

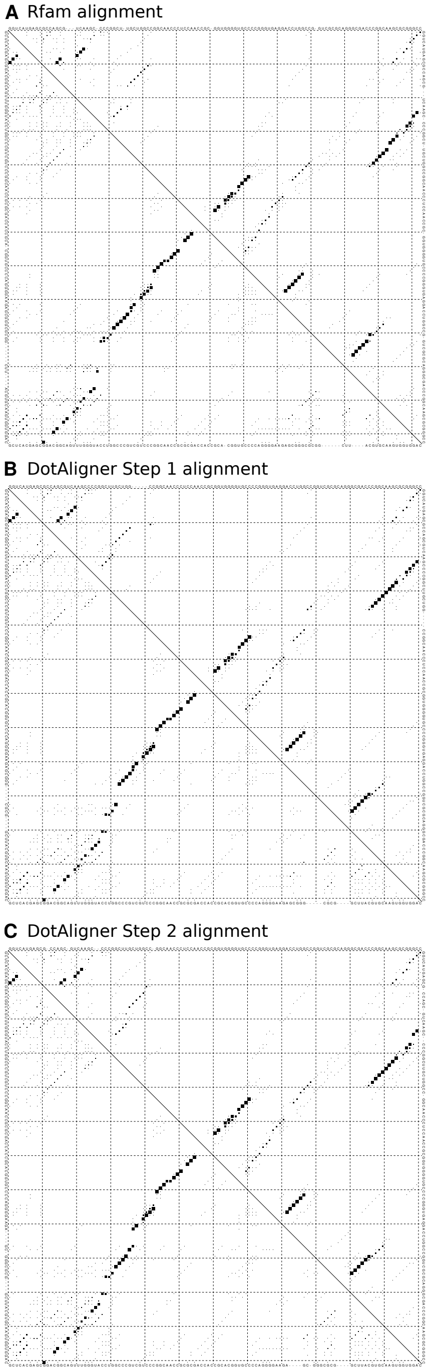

Figure S1. Pairwise alignments of dot plots of SAM riboswitch.
The two sequences AM420293_1 (upper triangles of the dot plots) and CP000580_2_6 (lower triangles) of the 5S-adenosyl methionine (SAM) riboswitch (Rfam family RF00634) are aligned (**A**) as in the Rfam reference alignment, (**B**) through `DotAligner`'s pairwise probabilistic string alignment (step 1), and (**C**) through `DotAligner`'s sampling of stochastic alignments (step 2). `DotAligner`'s sampling increases the combined score of the alignment from 0.58 to 0.60 (and the sequence identity from 56% to 63%), and improves the quality of the alignment compared to the Rfam reference.

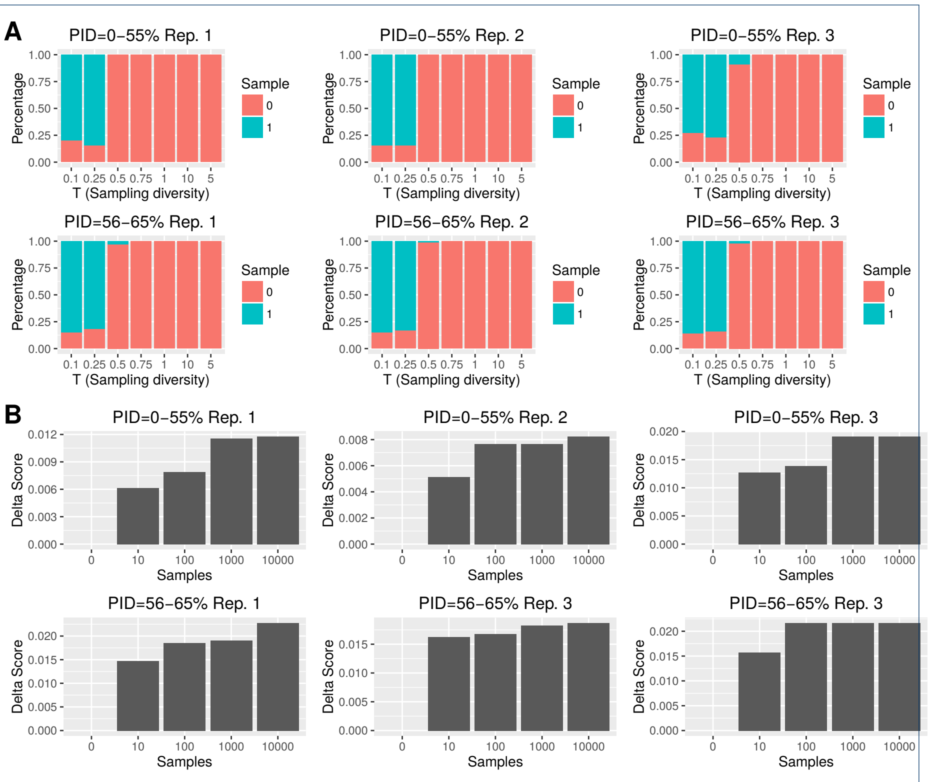

Figure S2. Impact of sampling of stochastic alignments on the alignment score. We executed `DotAligner` (default runtime parameters) while (**A**) varying parameter T (sampling diversity) with 1000 samples (parameter s) and (**B**) varying the number of samples with $T = 0.25$ on the RFAM binary classification benchmark datasets corresponding to 0-55% and 56-65% sequence identity (PID) (3 replicates each). For parameter T equal 0.1 and 0.25 the majority of pairwise alignments are optimized through the sampling procedure (sample increased the alignment score) (**A**). In few cases, T=0.5 also produced an optimized alignment through sampling. In average the alignment score saturates after 1000 samples of the stochastic backtracking for $T = 0.25$ (**B**).

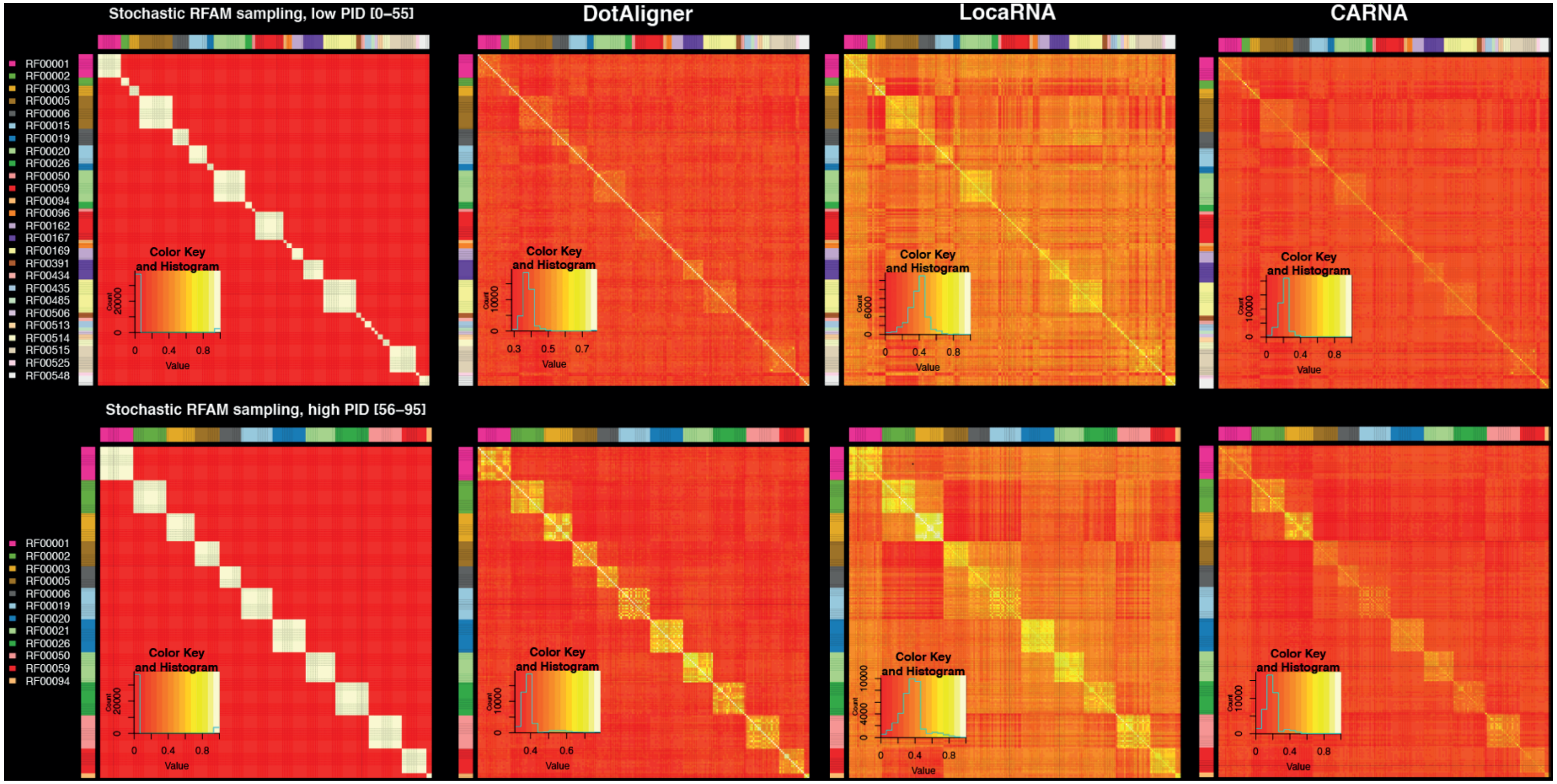

Figure S3. Binary classification of similarity matrices

Stochastically sampled RFAM version 12.0 sequences are labelled as belonging to the same family in white, and in red when not (Left). Heat maps of the similarity matrices produced by `DotAligner`, `LocaRNA` and `CARNA` are listed in columns 2, 3 and 4, respectively. (top) Low mean pairwise identity samples, where each sequence within a family shares between 0 and 55% sequence identity; (bottom) Higher (56-95%) mean pairwise identity samples.

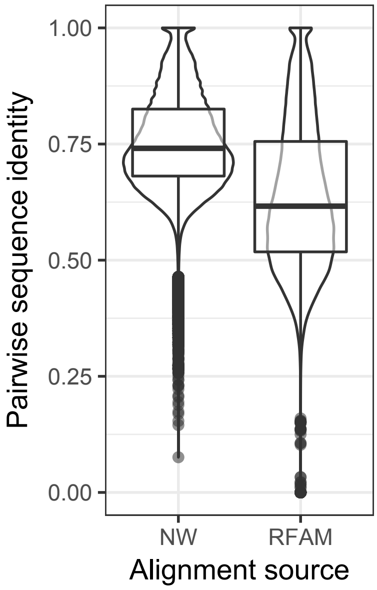

Figure S4. Difference in sequence identity between structural and sequence alignments

The difference in pairwise sequence identity for 1,189,675 randomly sampled RFAM version 12.3 seed alignments is shown for sequence-only alignments using a variant of the Needleman-Wunsch algorithm permitting free end gaps (NW) and the native RFAM seed alignments. Only sequences within the same family are compared, exposing the presence of local sequence similarity within the sequences. Pairwise sequence identity is defined by the number of matching nucleotides divided by the length of the shortest sequence.

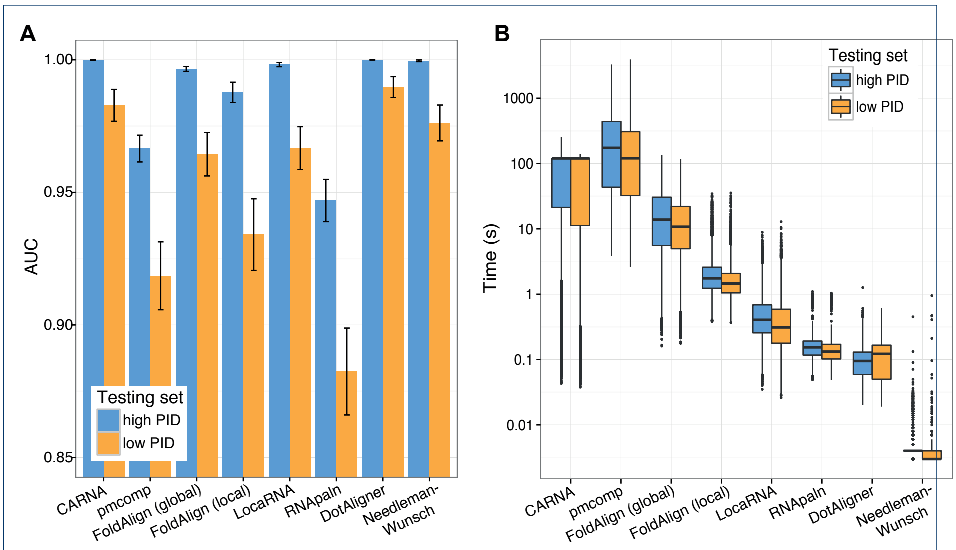

Figure S5. Classification of known RNA structures. (**A**) Area Under the Curve (AUC) of ROC values with 95% confidence intervals associated to Figure 3A. (**B**) Associated runtime distribution of single thread computation on a 2.6 GHz AMD Opteron processor (N.B. a fixed upper limit of 120 s was imposed for `CARNA`).

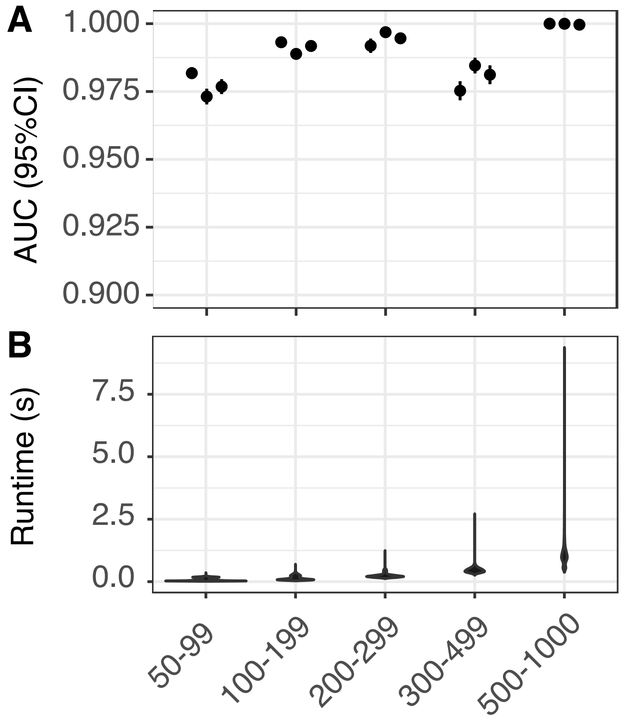

Figure S6. `DotAligner` clustering performance in function of sequence length. (**A**) Area Under the Curve (AUC) of ROC values with 95% confidence intervals for 3 replicates of stochastically sampled RFAM version 12.3 clans, controlling for sequence length (x-axis). N.B. the 500-1000 set only includes between 17-20 sequences given their rarity in the RFAM datasets, compared to 299-300 for the other samples. (**B**) Associated runtime distribution of single thread computation on a 2.6 GHz AMD Opteron processor.

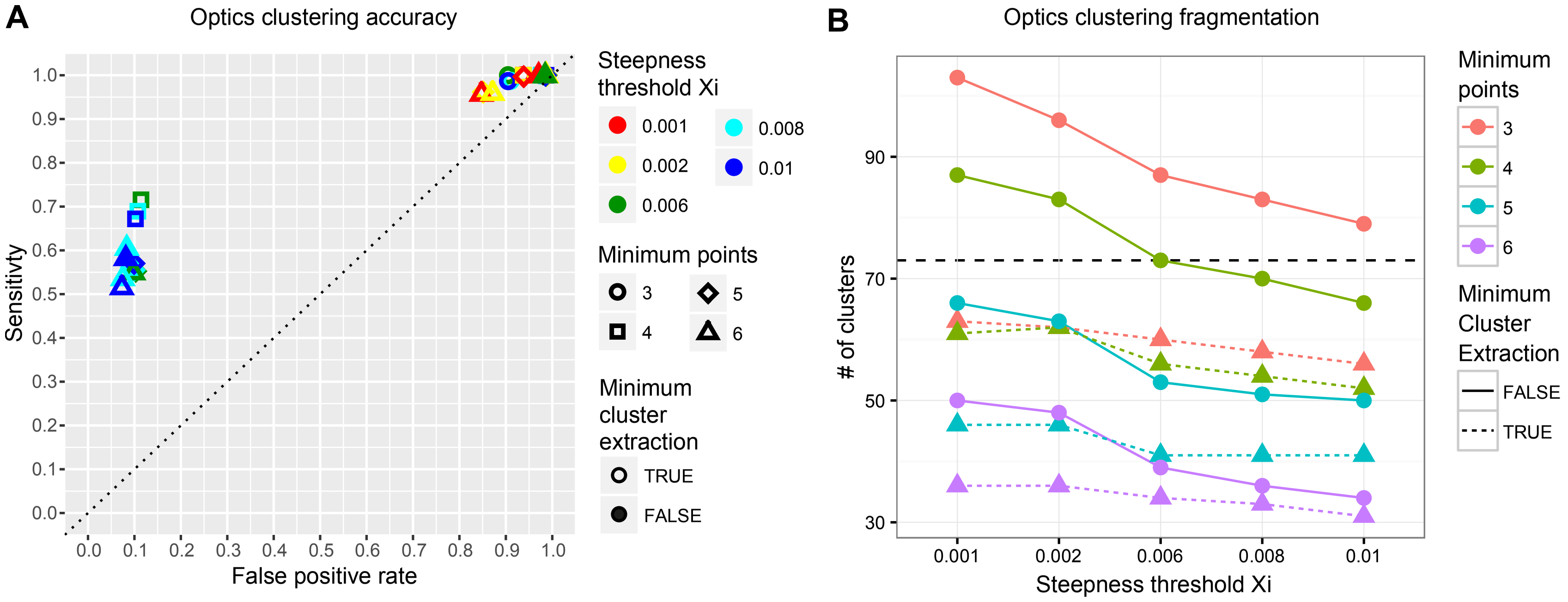

Figure S7. OPTICS clustering optimisation

Effect of OPTICS parameters on clustering accuracy (**A**) and amount of clusters (**B**) from a `DotAligner` dissimilarity matrix of 580 reference RFAM structures and their dinucleotide-shuffled controls (horizontal dashed line indicates expected amount of clusters, or unique RFAM families).

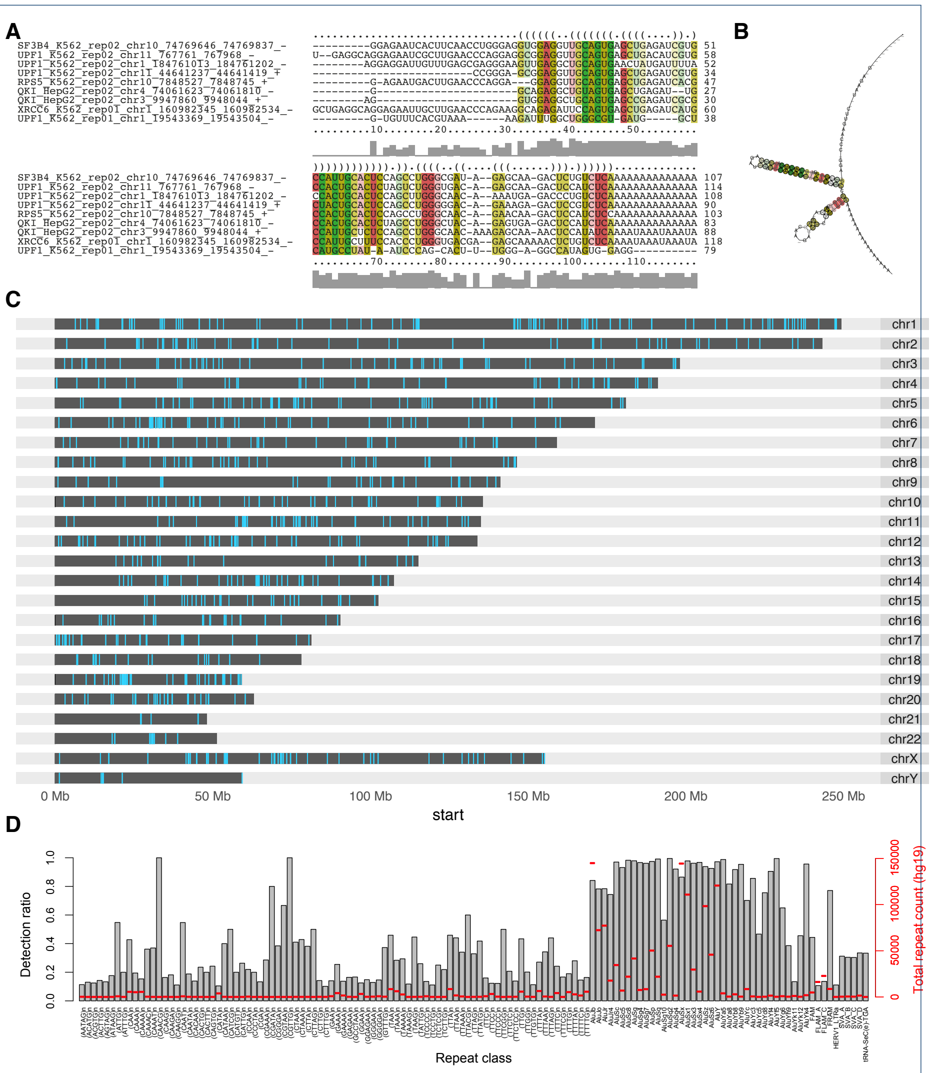

Figure S8. Genomic distribution of a UPF1-associated RNA structure motif
(**A**) Multiple sequence alignment of a significant cluster from **Figure 5F** as produced by `mLocaRNA` and `RNAalifold`, and its associated consensus secondary structure prediction (**B**). (**C**) Karyogram illustrating the human genomic coordinates (Grch37) of structural motif homologs, to this motif that do not overlap RepeatMasker annotations [?], as identified with `cmsearch` from the Infernal software package [49]. (**D**) Distribution of homologs within repeat elements (only repeats classes where > 10% of the repeats overlap homologs are displayed).
